# Der Ärztliche Leiter Führungsgruppe Katastrophenschutz als zentrale Entscheidungsinstanz bei der Steuerung regionaler Krankenhauskapazitäten in der Pandemie: Ein Erfahrungsbericht zur ersten Welle der COVID-19-Pandemie in einem bayerischen Regierungsbezirk

**DOI:** 10.1007/s00101-020-00911-6

**Published:** 2021-01-11

**Authors:** Michael S. Dittmar, Jürgen Altmeppen, Marc U. Bigalke, Florian Niedermirtl, Markus Zimmermann

**Affiliations:** 1 Ärztlicher Bezirksbeauftragter Rettungsdienst, Regierung der Oberpfalz, Sachgebiet 10, Emmeramsplatz 8, 93047 Regensburg, Deutschland; 2 Klinik für Anästhesiologie, Universitätsklinikum Regensburg, Regensburg, Deutschland; 3 Klinik für Anästhesie und operative Intensivmedizin, Klinikum Weiden, Weiden i. d. Oberpfalz, Deutschland; 4 Zentrale Notaufnahme, Klinikum St. Marien Amberg, Amberg, Deutschland; 5 Zweckverband für Rettungsdienst und Feuerwehralarmierung Amberg, Amberg, Deutschland; 6 Interdisziplinäre Notaufnahme, Universitätsklinikum Regensburg, Regensburg, Deutschland

**Keywords:** SARS-CoV-2, COVID-19, Katastrophenmedizin, Behandlungskapazitätenmanagement, Interhospitaltransfer, SARS-CoV-2, COVID-19, Disaster medicine, Surge capacity, Patient transfer

## Abstract

**Hintergrund und Zielsetzung:**

Von März bis Juni 2020 hatte Bayern die erste Welle der SARS-CoV-2-Pandemie zu bewältigen.

**Material und Methoden:**

Es werden Erfahrungen mit der Steuerung der stationären Behandlungskapazitäten für COVID-19-Patienten durch die Ärztlichen Leiter der Führungsgruppen Katastrophenschutz (ÄL-FüGK) und den Ärztlichen Bezirksbeauftragten Rettungsdienst (ÄBRD) in der Oberpfalz im Kontext des Notfallplan Corona-Pandemie der bayerischen Staatsregierung dargestellt.

**Ergebnisse:**

Durch Einstellen des Routineprogramms und Aufbau zusätzlicher Beatmungsbetten wurden Intensivkapazitäten geschaffen, welche insbesondere im Rettungsdienstbereich (RDB) Nordoberpfalz kurzfristig annähernd ausgelastet waren. Bei sich abzeichnendem Verlegungsbedarf von Intensivpatienten wählten die ÄL-FüGK bzw. der ÄBRD Zielkliniken im Sinne von Verlegungskorridoren aus. Dies erfolgte in drei eskalierenden Stufen: auf lokaler Ebene (RDB), auf regionaler Ebene (Regierungsbezirk) und auf überregionaler Ebene (zwischen Regierungsbezirken). Als Datengrundlage wurde u. a. die tägliche Bettenmeldung der Kliniken herangezogen. Normalstationskapazitäten waren stets frei, sodass Hilfskrankenhäuser nicht in Betrieb genommen werden mussten. Zum Schutz von Pflegeeinrichtungen verhängte die Staatsregierung einen Aufnahmestopp. Während des Abebbens der ersten Welle konnte die Routineversorgung schrittweise wieder aufgenommen werden.

**Diskussion:**

Die Steuerung der Patientenströme lehnte sich weitgehend an die Abläufe des Normalbetriebs an, was Abläufe verschlankte und Handlungsfähigkeit sicherstellte. Vereinzelt wurden Schnittstellenprobleme zu anderen Regierungsbezirken beobachtet, welche andere Managementgrundsätze verfolgten. Der Aufnahmestopp für Pflegeeinrichtungen und widerstreitende finanzielle Interessen der Klinikbetreiber stellten die ÄL-FüGK vor Herausforderungen.

## Abstract (en)

**Background and objective:**

During the initial phase of the COVID-19 pandemic the government of the state of Bavaria, Germany, declared a state of emergency for its entire territory for the first time in history. Some areas in eastern Bavaria were among the most severely affected communities in Germany, prompting authorities and hospitals to build up capacities for a surge of COVID-19 patients. In some areas, intensive care unit (ICU) capacities were heavily engaged, which occasionally made a redistribution of patients necessary.

**Material and methods:**

For managing COVID-19-related hospital capacities and patient allocation, crisis management squads in Bavaria were expanded by disaster task force medical officers (“Ärztlicher Leiter Führungsgruppe Katastrophenschutz” [MO]) with substantial executive authority. The authors report their experiences as MO concerning the superordinate patient allocation management in the district of Upper Palatinate (Oberpfalz) in eastern Bavaria.

**Results:**

By abandoning routine patient care and building up additional ICU resources, surge capacity for the treatment of COVID-19 patients was generated in hospitals. In parts of the Oberpfalz, ICU capacities were almost entirely occupied by patients with corona virus infections, making reallocation to other hospitals within the district and beyond necessary. The MO managed patient pathways in an escalating manner by defining local (within the region of responsibility of a single MO), regional (within the district), and cross-regional (over district borders) reallocation lanes, as needed. When regional or cross-regional reallocation lanes had to be established, an additional management level located at the district government was involved. Within the determined reallocation lanes, emitting and receiving hospitals mutually agreed on any patient transfer without explicitly involving the MO, thereby maintaining the established interhospital routine transfer procedures. The number of patients and available treatment resources at each hospital were monitored with the help of a web-based treatment capacity registry. If indicated, reallocation lanes were dynamically revised according to the present situation. To oppose further virus spreading in nursing homes, the state government prohibited patient allocation to these facilities, which led to considerably longer hospital length of stay of convalescent elderly and/or dependent patients. In parallel to the flattening of the COVID-19 incidence curve, routine hospital patient care could be re-established in a stepwise manner.

**Conclusion:**

Patient allocation during the state of emergency by the MO sought to keep up routine interhospital reallocation procedures as much as possible, thereby reducing management time and effort. Occasionally, difficulties were observed during patient allocations crossing district borders, if other MO followed different management principles. The nursing home blockade and conflicting financial interests of hospitals posed challenges to the work of the disaster task force medical officers.

## Hintergrund

Deutschland, wie die gesamte Welt, erlebt zurzeit aufgrund der von SARS-CoV‑2 („severe acute respiratory syndrome coronavirus 2“) ausgelösten Pandemie, die zur „coronavirus disease 2019“ (COVID-19) genannten Erkrankung führt, die wohl schwerste Krisensituation seit dem Zweiten Weltkrieg. Von der Feststellung des ersten bundesdeutschen COVID-19-Falls im bayerischen Landkreis Starnberg Ende Januar 2020 bis in die folgenden Monate wurden durch entsprechende Verordnungen der Bundesländer sowie durch Änderungen des Infektionsschutzgesetzes zahlreiche Maßnahmen zum Schutz der Bevölkerung ergriffen. Ziel der Maßnahmen war insbesondere, bei einem zunächst exponentiellen Verlauf der COVID-19-Inzidenz, eine Überlastung des Gesundheitssystems angesichts besonders schwerer und lebensbedrohlicher Krankheitsverläufe und Erfahrungen mit erheblichen Engpässen, wie zuvor in anderen Ländern geschehen [15, 18, 23, 24, 31], zu vermeiden.

Um die Krankenhäuser auf die zu erwartenden massiven Fallzahlsteigerungen vorzubereiten, verpflichtete das Bayerische Staatsministerium für Gesundheit und Pflege (StMGP) die Krankenhäuser durch Allgemeinverfügung vom 19.03.2020, soweit medizinisch vertretbar, alle elektiven Behandlungen zurückzustellen oder zu unterbrechen sowie gleichzeitig ihre vorhandenen Kapazitäten auszubauen und für die Versorgung von COVID-19- und anderen Notfallpatienten in vollem Umfang zur Verfügung zu stellen [8]. Vor diesem Hintergrund hatte die bayerische Staatsregierung aufgrund der COVID-19-Pandemie am 16.03.2020 den Katastrophenfall für Bayern ausgerufen [5]. Für die Organisation und Steuerung der Krankenhauskapazitäten wurde mit einer weiteren Allgemeinverfügung mit Wirkung zum 25.03.2020 in jedem der 26 bayerischen Rettungsdienstbereiche (RDB) die Funktion eines Ärztlichen Leiters Führungsgruppe Katastrophenschutz (ÄL-FüGK) mit weitreichenden Kompetenzen etabliert [6].

Im folgenden Bericht stellen die Autoren ihre Erfahrungen mit der Steuerung der stationären Behandlungskapazitäten für COVID-19-Patienten durch die ÄL-FüGK im Regierungsbezirk Oberpfalz, Bayern, während der ersten Welle der COVID-19-Pandemie dar.

### Geografie der Oberpfalz

Die ländlich geprägte Oberpfalz (Fläche ca. 9700 km^2^) liegt im Nordosten Bayerns und ist einer von 7 Regierungsbezirken, welcher sich in 3 kreisfreie Städte und 7 Landkreise gliedert und in dem rund 8,5 % der ca. 13 Mio. Einwohner Bayerns leben. Die Region zählte zu den „Hotspots“ der Coronapandemie in Bayern, insbesondere der Landkreis Tirschenreuth, welcher die bundesweit höchste COVID-19-Inzidenz zu bewältigen hatte [29, 30].

Regensburg bildet die Hauptstadt des Regierungsbezirks. Die Stadt hat ca. 153.000 Einwohner und steht damit nach München, Nürnberg und Augsburg an 4. Stelle unter den Großstädten Bayerns. Weitere große Städte in der Oberpfalz sind Weiden i. d. Opf. und Amberg mit je rund 40.000 Einwohnern.

Die bayerischen Landkreise und kreisfreien Städte (Gebietskörperschaften) stellen in kommunaler Zusammenarbeit durch Zweckverbände für Rettungsdienst und Feuerwehralarmierung (ZRF) den öffentlichen Rettungsdienst sicher. Die Gebietskörperschaften sind gemäß des Bayerischen Rettungsdienstgesetzes (Art. 4 BayRDG i. V. m. § 1 AVBayRDG) einem von 26 RDB zugeordnet. Die Städte Regensburg, Weiden und Amberg bilden jeweils zusammen mit den umliegenden Landkreisen entsprechend 3 RDB, die je eine Integrierte Leitstelle (ILS) zur Einsatzlenkung vorhalten. Insgesamt wurden im Jahr 2019 in den 3 genannten RDB ca. 164.000 Rettungsdienstereignisse (Notfallereignisse, Krankentransporte und arztbegleitete Patiententransporte) dokumentiert [21].

### Katastrophenschutz in Bayern

Katastrophenschutz ist eine staatliche Aufgabe in der Zuständigkeit der Bundesländer, der von den Katastrophenschutzbehörden wahrgenommen wird. In Bayern sind die Katastrophenschutzbehörden die Kreisverwaltungsbehörden, also Landratsämter und kreisfreie Städte, die Bezirksregierungen sowie das Bayerische Staatsministerium des Innern, für Sport und Integration (StMI). Zur Bewältigung der Aufgaben im Katastrophenfall bedienen sich die Katastrophenschutzbehörden einer Führungsgruppe Katastrophenschutz (FüGK). Sie setzt sich grundsätzlich aus Mitarbeitern der Behörde zusammen und wird bei Bedarf lageabhängig durch Vertreter anderer betroffener Behörden und Einrichtungen, durch Vertreter der an der Katastrophenbewältigung beteiligten Einsatzorganisationen und durch Sachverständige erweitert (Art. 2, 5 BayKSG).

### Krankenhausvorhaltung in der Oberpfalz

**Figure 1:**
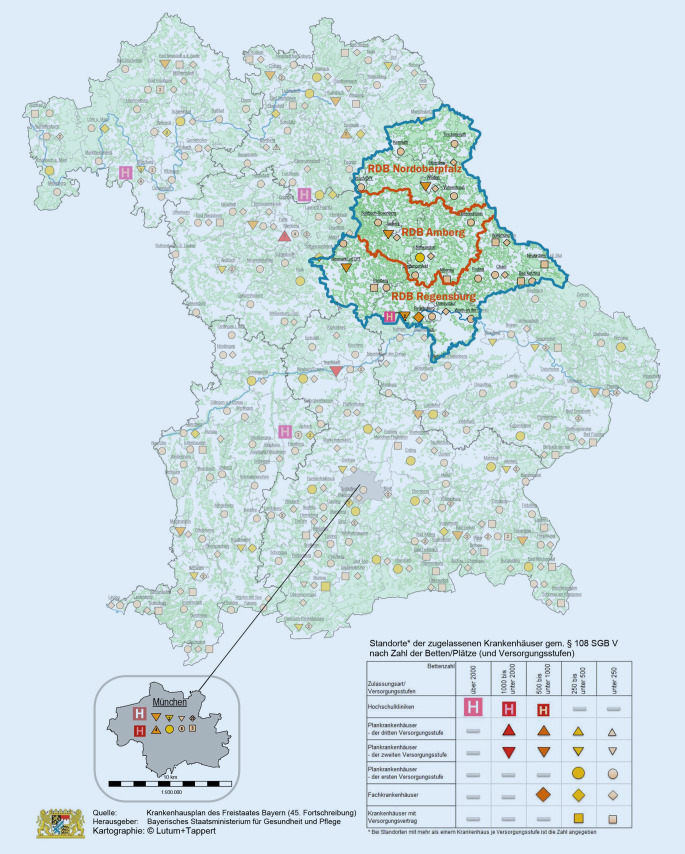

Im Gebiet der Oberpfalz befinden sich insgesamt 28 Krankenhäuser mit Versorgungsvertrag (eine Hochschulklinik, 5 Krankenhäuser der Versorgungsstufe II, 14 Krankenhäuser der Versorgungsstufe I und 8 Fachkliniken) mit zusammen über 6400 Planbetten [9]. Einen Überblick über die Oberpfalz, ihre Landkreise und Städte, Rettungsdienstbereiche, sowie die Anzahl und Versorgungsstufe der Krankenhäuser zeigt Abb. 1.

## Material und Methoden

Die Autoren berichten über Organisationsstruktur und Abläufe des stationären Bettenkapazitätsmanagements und der Steuerung der Patientenströme sowie die daraus gewonnenen Erfahrungen aus Sicht der ÄL-FüGK und des ÄBRD im Regierungsbezirk Oberpfalz im Kontext der behördlichen Anordnungen zum COVID-19-Krisenmanagement.

Die Daten zur Bettensituation in den Oberpfälzer Krankenhäusern wurden bis zum 14.04.2020 den verpflichtenden täglichen Bettenmeldungen der Kliniken an die Katastrophenschutzbehörden entnommen, ab 15.04.2020 dem webbasierten interdisziplinären Versorgungsnachweis IVENA eHealth Sonderlage COVID-19 (Fa. mainis IT-Service GmbH, Offenbach am Main), welcher ebenfalls von den Kliniken verpflichtend gepflegt wurde.

## Ergebnisse

### Organisation der Krankenhausbelegung

Im Rahmen des Katastrophenmanagements und auf Grundlage des Infektionsschutzgesetzes haben am 24.03.2020, also eine Woche nach dem Ausrufen des Katastrophenfalls in Bayern, das StMGP und das StMI den „Notfallplan Corona-Pandemie“ zur Bewältigung erheblicher Patientenzahlen in Krankenhäusern in Form einer Allgemeinverfügung erlassen [6].

Wesentlicher Inhalt der Allgemeinverfügung waren zentrale Vorgaben zu Organisation und Steuerung der Krankenhausbelegung. Ziel war es, über die Steuerung der Patientenströme und die Belegung der Krankenhauskapazitäten mit COVID-19-Patienten möglichst dezentral innerhalb der regionalen Krankenhausstrukturen zu entscheiden. Es sollten alle geeigneten Maßnahmen ergriffen werden, um die Krankenhäuser auf die erwartete massive Fallzahlsteigerung vorzubereiten und die Patientenströme so geordnet und effizient wie möglich zu lenken.

Hierzu wurde für jeden RDB die Funktion eines AL-FüGK mit umfassenden Weisungsrechten eingesetzt. Der ÄL-FüGK hatte die Aufgabe, das Betten- und Behandlungskapazitätenmanagement sowie die Patientenströme aller stationären Einrichtungen in der jeweiligen Versorgungsregion für die Bekämpfung der Coronapandemie übergeordnet zu steuern. Zu seinen Aufgaben gehörte insbesondere die Benennung für die Behandlung von COVID-19-Patienten vorrangig zuständiger Krankenhäuser, sog. COVID-19-Schwerpunktkrankenhäuser (Tab. 1). Die Ernennung des ÄL-FüGK erfolgte durch den vorsitzenden Landrat bzw. Oberbürgermeister des ZRF, wobei als Mindestqualifikation die klinische Tätigkeit in der Akutmedizin, die fachliche Expertise für Krisenbewältigung sowie mindestens die Funktion als Oberarzt vorgegeben waren. Vielerorts griffen die ZRF auf den lokalen Ärztlichen Leiter Rettungsdienst (ÄLRD) für diese Aufgabe zurück. In der Oberpfalz wurden 2 ehemalige ÄLRD, welche heute jeweils als Leiter einer interdisziplinären Notaufnahme wirken, sowie ein Chefarzt einer Anästhesieabteilung ernannt.

**Table 1:** 

Übergeordnete Steuerung des COVID-19 Betten- und Behandlungskapazitätenmanagements sowie der Patientenströme aller Einrichtungen	
Mitglied und Integration in die Struktur Führungsgruppe Katastrophenschutz (FüGK) mit fachlichem Weisungsrecht im Aufgabenbereich (einschließlich aller anderen im Versorgungsbereich bestehenden FüGK (untere Katastrophenschutzbehörden)) *z.* *B. Überwachung und Freigabe der bei den FüGK angemeldeten zusätzlichen Materialbedarfe*	
Bestimmung der für die Behandlung von COVID-19-Patienten vorrangig zuständigen Krankenhäuser (COVID-19-Schwerpunktkrankenhäuser)	
Laufende Sichtung der durch die Krankenhäuser verpflichtend gepflegten IVENA-Sonderlage	
Abstimmung mit	– der Integrierten Leitstelle (ILS)
	– den Ärztlichen Leitern Rettungsdienst (ÄLRD) im Bereich des Rettungsdienstes
	– den Pandemiebeauftragten der Krankenhäuser (*z.* *B. Festlegung der Screeningabläufe, Verlagerung von Ausstattung und Personal u.* *a.)*
	– der COVID-19-Koordinierungsgruppe Krankenhaus inkl. Weisungsrecht und
	– den Ärztlichen Bezirksbeauftragten Rettungsdienst (ÄBRD)
Befugnis zur Ergreifung geeigneter Maßnahmen zur ärztlichen Besetzung des Notarztdienstes bei Engpässen (in Abstimmung mit ÄLRD)	

Zur möglichst effizienten Abstimmung unter den Krankenhäusern waren grundsätzlich auf Ebene der Kreisverwaltungsbehörden COVID-19-Koordinierungsgruppen der Kliniken einzurichten, die mit den Pandemiebeauftragten der Einrichtungen zu besetzen waren. Je nach regionaler Notwendigkeit konnten mit Zustimmung des ÄL-FüGK auch anders zugeschnittene Koordinierungsbereiche gebildet werden. Ein Sprecher der Koordinierungsgruppe sollte Ansprechpartner der COVID-19-Koordinierungsgruppe für Dritte sein und stand insbesondere für den Austausch und die Zusammenarbeit mit den ILS und dem ÄL-FüGK zur Verfügung. Der ÄL-FüGK, die Kreisverwaltungsbehörde, das Gesundheitsamt, der ÄLRD sowie die örtlichen Vertreter der kassenärztlichen Vereinigung konnten an den Sitzungen der COVID-19-Koordinierungsgruppe teilnehmen. Der ÄL-FüGK hatte gegenüber der COVID-19-Koordinierungsgruppe ein Weisungsrecht; er konnte Entscheidungen der COVID-19-Koordinierungsgruppe ändern oder aufheben. Zudem sollte der ÄL-FüGK sich eng mit der ILS, den ÄLRD für den Bereich des Rettungsdienstes und der oben genannten COVID-19-Koordinierungsgruppe Krankenhaus abstimmen.

**Figure 2:**
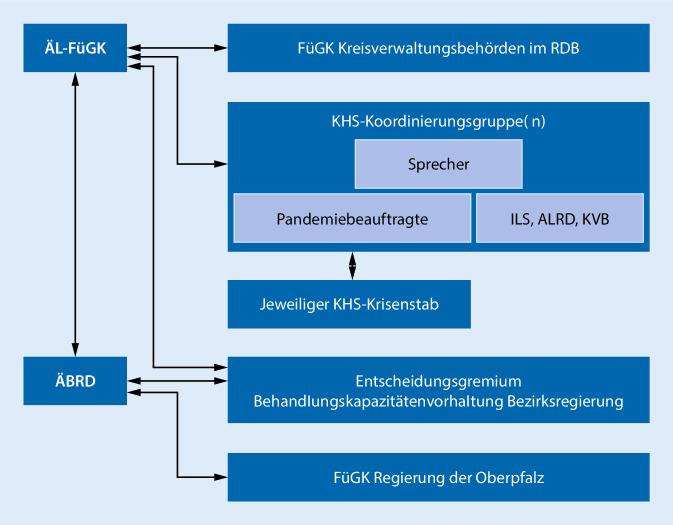

Zur Unterstützung und zur Koordination der ÄL-FüGK im Rahmen der überregionalen Steuerung der Patientenströme bediente sich der Notfallplan Corona-Pandemie der Ärztlichen Bezirksbeauftragten Rettungsdienst (ÄBRD) bei den einzelnen Bezirksregierungen [6]. Im Regelbetrieb koordiniert und beaufsichtigt der ÄBRD die ÄLRD seines Zuständigkeitsbereichs und übernimmt die überregionale Gremienarbeit und Steuerung des Qualitätsmanagements im Rettungsdienst (Art. 12 Abs. 2 BayRDG). Die Verantwortlichkeiten aus dem Notfallplan stellten somit für den ÄBRD eine zusätzliche Sonderaufgabe im Auftrag der Regierung dar. Einen Überblick über die Führungsstruktur des Bettenkapazitätenmanagements gibt Abb. 2.

**Figure 3:**
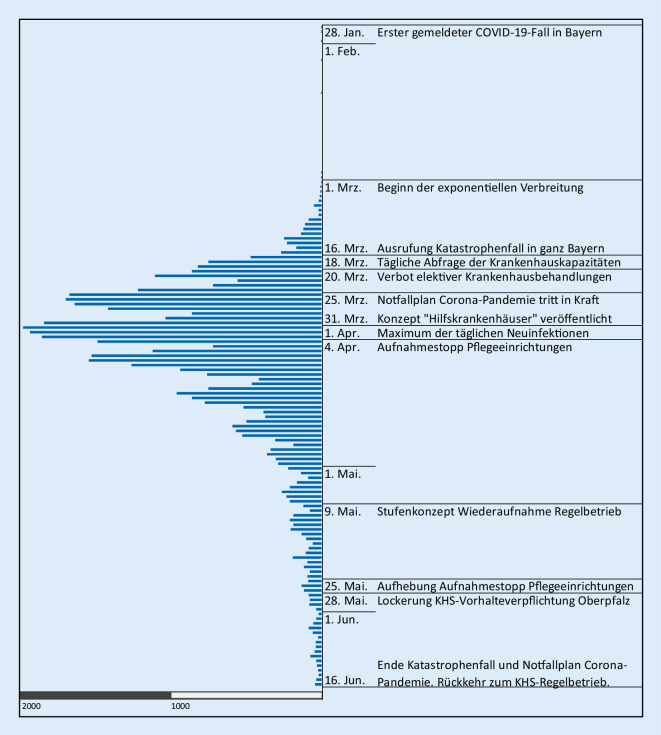

Bereits zuvor waren mit der täglichen Abfrage der Krankenhauskapazitäten, zunächst über Tabellenverarbeitung, und die Verschiebung elektiver Krankenhausbehandlungen zur Schaffung zusätzlicher Kapazitäten erste Vorkehrungen zur Krisenbewältigung getroffen worden ([8]; Abb. 3).

### Krisenvorbereitung der Krankenhäuser

Eines der wichtigsten Ziele des Notfallplans Corona-Pandemie war die Schaffung ausreichender Krankenhausbehandlungskapazitäten für die befürchtete hohe Zahl an COVID-19-Patientinnen und -Patienten. Dies erfolgte neben der weitestgehenden Aussetzung der elektiven Patientenversorgung [8] durch die Verpflichtung zur Schaffung zusätzlicher Bettenkapazitäten, insbesondere von Beatmungsbetten auf Intensivstationen, zusammen mit der notwendigen Ausstattung und entsprechendem Personal. Innerhalb der Kliniken wurde je ein Krisenstab gefordert und für Absprachen nach außen ein Pandemiebeauftragter benannt. Die jeweils verfügbaren Kapazitäten waren mindestens einmal täglich über das Online-Softwaretool IVENA eHealth Sonderlage zu aktualisieren [6].

Nicht anderweitig zu deckende Bedarfe an Ausstattung, Material und Personal sollten einerseits über einen Austausch der Kliniken untereinander, andererseits durch Lieferungen von Medizintechnik durch Land und Bund bzw. die Vermittlung von Freiwilligen über einen sogenannten Pflegepool bedient werden.

Daneben setzten die Kliniken noch weitere Infektionsschutzvorkehrungen im Sinne von Distanzierungsmaßnahmen (Lenkung von Patienten- und Besucherströmen, Kohortendienstpläne), Teststrategien und Isolationsvorkehrungen für bestätigte COVID-19- und Verdachtsfälle um. Bei vielen dieser Maßnahmen war auch der ÄL-FüGK involviert [6].

Hervorzuheben ist, dass nicht nur Akutkliniken aus dem Krankenhausbedarfsplan in die oben skizzierte Vorbereitung einbezogen wurden, sondern gleichermaßen auch sonstige Krankenhäuser mit Versorgungsvertrag (§ 109 SGB V), Einrichtungen der Vorsorge und Rehabilitation sowie Privatkliniken mit Zulassung nach § 30 GewO [6].

### Ablauforganisation des Behandlungskapazitätenmanagements

**Figure 4:**
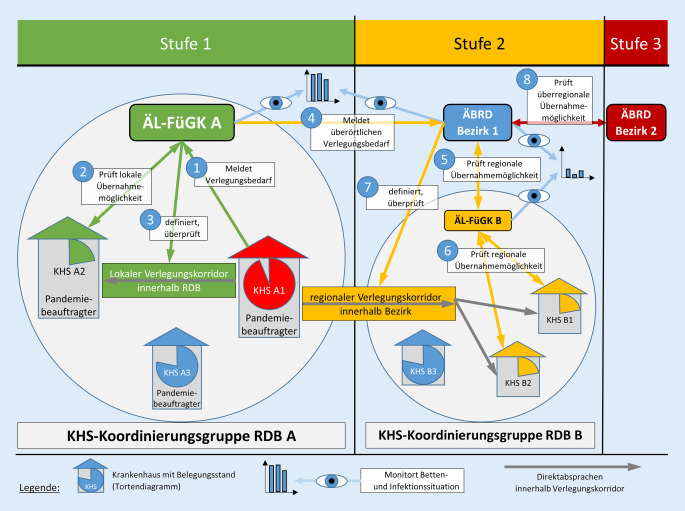

Für die Oberpfalz vereinbarten ÄL-FüGK und ÄBRD ein mehrstufig gegliedertes Bettenkapazitätenmanagement (Abb. 4). Die Verantwortlichen wurden in diesem Zusammenhang hauptsächlich im Rahmen von Verlegungen tätig, z. B. bei der Entlastung von Intensivkapazitäten oder bei Verlegungsbedarf aus medizinischen Gründen. Nur in Ausnahmefällen wurde organisatorisch bei der primären Aufnahme von Notfallpatienten unterstützt. Zunächst stand der Bedarf an intensivmedizinischer Versorgung im Vordergrund, in der Spätphase der ersten Welle die Suche nach geeigneten Abverlegungsmöglichkeiten für postakute COVID-19-Patienten, die häufig noch einen positiven SARS-CoV-2-Nachweis mitbrachten.

Bei der Steuerung der Patientenströme erfolgte, soweit wie möglich, ein Rückgriff auf etablierte Strukturen und Abläufe aus der Routinesituation. Statt das Verlegungsmanagement auf der Ebene einzelner Patienten zu betreiben, wurden sog. Verlegungskorridore bestimmt, innerhalb derer die abgebenden Kliniken selbstständig den direkten Kontakt zur potenziell aufnehmenden Klinik aufnahmen und die Verlegungsmodalitäten abstimmten. Die Bettensituation wurde von den ÄL-FüGK/ÄBRD kontinuierlich über IVENA beobachtet und die Verlegungskorridore bei Bedarf angepasst. Hierdurch konnte eine weitgehend friktionsfreie Kombination der bekannten und bewährten Verlegungsabläufe mit einer übergeordneten Lenkung der Patientenströme erreicht werden.

Der zuständige ÄL-FüGK wurde durch den Pandemiebeauftragten oder durch eigene Beobachtung auf konkreten oder antizipierten Verlegungsbedarf aufmerksam. Daraufhin klärte der ÄL-FüGK zunächst im eigenen ZRF-Bereich geeignete Übernahmemöglichkeiten ab und nannte der abgebenden Klinik eine oder mehrere zu kontaktierende Kliniken im Sinne eines RDB-internen Verlegungskorridors (*Stufe 1*, in Abb. 4). Sofern dies nicht erfolgreich war, erfolgte eine Kontaktaufnahme zum ÄBRD, der wiederum mit dem ÄL-FüGK eines anderen ZRF-Gebiets im eigenen Bezirk passende Aufnahmekliniken vereinbarte (RDB-übergreifender Verlegungskorridor, *Stufe 2* in Abb. 4). Die Festlegung der genauen Anzahl, die Auswahl und der jeweilige Verlegungszeitpunkt der zu verlegenden Patienten wurden wiederum im direkten Kontakt der Kliniken vereinbart. Bei notwendigen Verlegungen über die Grenzen des Regierungsbezirks hinweg fand eine entsprechende Absprache zwischen den beiden zuständigen ÄBRD statt (*Stufe 3* in Abb. 4).

Zeitkritische Abverlegungen oder Aufnahmen führten die Kliniken zudem zu jedem Zeitpunkt ohne Rücksprache mit dem ÄL-FüGK, ggf. auch abseits der vorgesehenen Verlegungskorridore, durch. Der jeweils zuständige ÄL-FüGK erhielt in diesen Fällen eine nachträgliche Information über den Vorgang. Hierdurch wurde die ständige unmittelbare Handlungsfähigkeit der Krankenhäuser in Notfällen gewährleistet.

### Verlauf der Infektions- und Bettensituation

Die höchste COVID-19-Falldichte in der Oberpfalz war im Rettungsdienstbereich Nordoberpfalz zu verzeichnen, allen voran im Landkreis Tirschenreuth mit einer kumulativen Inzidenz von 1572 Fällen/100.000 Einwohner, gefolgt vom Landkreis Neustadt a. d. Waldnaab mit 892 und der Stadt Weiden i. d. Opf. mit 797 Fällen/100.000 Einwohner (Stand 29.07.2020) [3]. Hier kam es kurzfristig zu einer annähernden lokalen Vollauslastung der Intensivstationen und konsekutivem Verlegungsbedarf aus Kapazitätsgründen, zusätzlich zu medizinisch indizierten Patiententransfers. Intensivmedizinische Reservekapazität war jedoch zu jedem Zeitpunkt vorhanden. Die sonstigen Oberpfälzer Gebietskörperschaften wiesen bis Ende Juli kumulative bevölkerungsbezogene Fallzahlen zwischen 226 (Stadt Amberg) und 481 (Landkreis Amberg-Sulzbach) auf [3].

**Figure 5:**
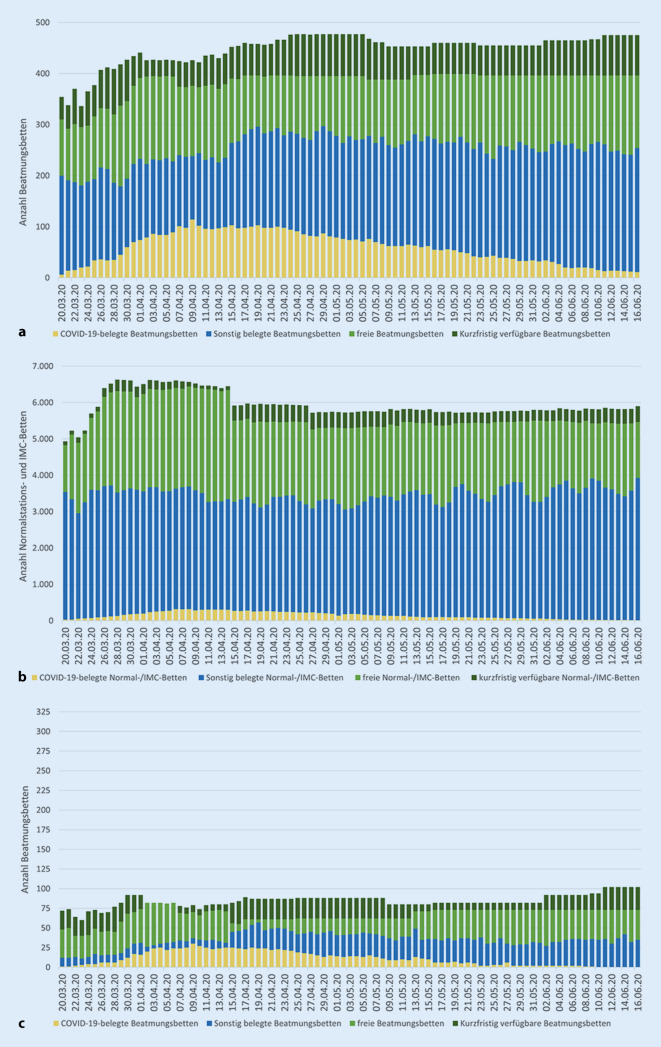

Während die Intensivstationen im RDB Nordoberpfalz vorübergehend eine hohe Auslastung aufwiesen, konnten die sonstigen Krankenhäuser der Oberpfalz stets ausreichend freie Intensivbehandlungskapazitäten vorweisen. Neben der Deckung des Oberpfälzer Bedarfs konnten insbesondere am Universitätsklinikum Regensburg auch einzelne Patientinnen und Patienten aus anderen Regierungsbezirken mit dem Schwerpunkt Oberbayern sowie aus Italien versorgt werden. Gleichzeitig waren bezirksweit bis zu 3101 freie Normalstationsbetten gemeldet. Die COVID-19-bezogenen Betten- und Belegungssituationen der Kliniken in den Oberpfälzer RDB sind Abb. 5 zu entnehmen.

**Figure 6:**
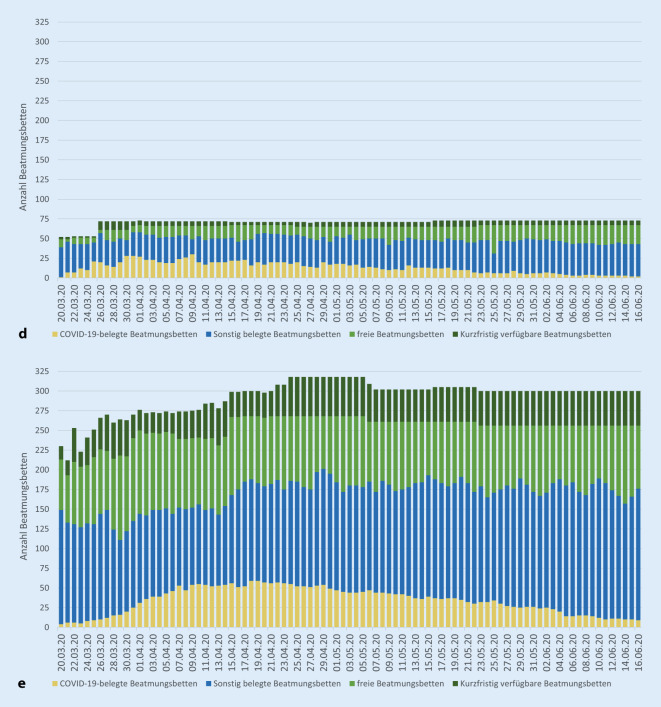

Im Zuge der Umsetzung des Notfallplans Corona-Pandemie konnten in den Akutkrankenhäusern der Oberpfalz zusätzliche Bettenkapazitäten in der Größenordnung von ca. 50 % der Regelbeatmungsbetten bzw. 25 % der sonstigen Betten geschaffen werden (Tab. 2). Zur Ausstattung der neu geschaffenen Isolier- und Intensivbettenkapazität der Oberpfälzer Akutkrankenhäuser stellten die Bundes- und Landesregierung Medizingeräte und/oder Material zur Verfügung.

**Table 2:** 

	Beatmungsbetten				Normal- und IMC-Betten			
	Regelbetrieb	Maximale Vorhaltung	Steigerung (%)	Maximale COVID-19-Belegung	Regelbetrieb	Maximale Vorhaltung	Steigerung (%)	Maximale COVID-19-Belegung
RDB Amberg	48	102 (11.06.)	113	30 (09.04.)	1431	1588 (28.03.)	11	76 (06.04.)
RDB Nordoberpfalz	49	73 (01.04.)	49	30 (09.04.)	812	1124 (25.05.)	38	150 (06.04.)
RDB Regensburg	213	318 (24.04.)	49	59 (18.04.)	2999	4098 (29.03.)	37	106 (08.04.)
Oberpfalz	310	477 (25.04.)	54	114 (09.04.)	5242	6629 (28.03.)	27	319 (08.04.)

Als Basiswert für den Regelbetrieb wurde die erste Bettenmeldung an die Behörden im Zeitraum 20.–23.03.2020 herangezogen. Angaben für die gesamte Oberpfalz geben die jeweils gleichzeitig bestehende Situation wieder, nicht die Summe der sonstigen Tabellenzeilen. Die Datumsangabe bezeichnet den jeweils ersten Tag des Höchststandes

*RDB* Rettungsdienstbereich, *IMC* „intermediate care“

*Quelle*: Bettenmeldungen an die FüGK der Regierung, IVENA Sonderlage

Neben Engpässen bei persönlicher Schutzausrüstung kam es insbesondere zu einer angespannten Versorgungssituation bei intensivmedizinischen Medikamenten wie z. B. Sedativa und Katecholaminen. Die Verteilung der Lieferungen war meist auf Basis des letztjährigen Routineverbrauchs vorgesehen, was jedoch in besonders mit COVID-19-Patienten belasteten Kliniken den tatsächlichen aktuellen Bedarf nicht decken konnte. Die ÄL-FüGK haben hierzu eine Anpassung des Verteilungsschlüssels an die lokale Infektionsaktivität gefordert, welche momentan zur Diskussion steht.

Für Krankenhäuser, welche viel Personal aus dem benachbarten Ausland beschäftigen, stellte sich die Aussetzung der Fachsprachenprüfungen [1] als Problem für die Personalrekrutierung im ärztlichen Dienst heraus. Erfreulicherweise macht die zuständige Regierung von Oberbayern nach einer Anregung aus den Krisenstäben großzügig von der Möglichkeit des § 10 Abs. 1a BÄO Gebrauch, welcher das Aussprechen einer auf ein Jahr befristeten Berufserlaubnis bei besonderem Interesse bereits vor der vorliegenden Sprachprüfung ermöglicht. Hierdurch konnten Personalengpässe abgemildert werden.

### Hilfskrankenhäuser

Parallel zur Ausweitung der Versorgungskapazitäten an den bestehenden Krankenhäusern strengten einige FüGK bereits in der Frühphase des Katastrophenfalls Planungen und Vorbereitungen zu Errichtung von Hilfskrankenhäusern zur Entlastung evtl. überlasteter Kliniken an. Ende März gab das StMI dann ein einheitliches Konzept für Hilfskrankenhäuser heraus, an dem sich weitere Planungen orientierten. In der Oberpfalz musste jedoch letztlich kein Hilfskrankenhaus in Betrieb genommen werden.

### Rettungsdienstliche Vorkehrungen und Notarztdienstbesetzung

Entgegen anfänglicher Befürchtungen war keine flächendeckende Überlastung des Rettungsdienstes zu beobachten. Allerdings ist bei der Bewertung der Einsatzzahlen zu berücksichtigen, dass die Umsetzung der umfassenden Hygienekonzepte zu verlängerten Einsatzzeiten führte. Es wurden auch keine der befürchteten Besetzungsprobleme an Oberpfälzer Notarztstandorten an die ÄL-FüGK herangetragen. Daher gab es keine Veranlassung für die ÄL-FüGK, die ihnen gegenüber Ärztinnen und Ärzten sowie den Krankenhausträgern übertragenen Befugnisse zur Sicherstellung der Notarztdienstbesetzung auszuüben [6].

Um einen antizipierten erhöhten Verlegungsbedarf von Intensivpatienten bedienen zu können, ordnete das StMI im Rahmen des Notfallplans Corona-Pandemie die gerätetechnische Ertüchtigung eines Rettungstransportwagens (RTW) pro RDB zu einem Verlegungs-RTW (V-RTW) an. Die Arztbesetzung dieser subsidiären Einsatzmittel für Intensivtransporte war durch Verlegungsärzte nach Art. 15 Abs. 2 BayRDG oder sonstige intensivmedizinisch erfahrene, niedergelassene oder Krankenhausärzte vorgesehen.

Qualifizierte Krankentransporte von COVID-19-(Verdachts‑)Fällen sollten vorwiegend von einzelnen, hierzu aus der Regelvorhaltung vorab ausgewählten Krankentransportwägen („KTW-Corona“) durchgeführt werden.

### Aufnahmestopp in Pflegeeinrichtungen

Eine Reihe von Alten- und Pflegeeinrichtungen im Bezirk musste eine nichtunerhebliche Zahl an Infektions- und auch Todesfällen verzeichnen. Zum Schutz der vulnerablen Population in Alten- und Pflegeheimen untersagte das StMGP zum 04.04.2020 die Neu- und Wiederaufnahme von Bewohnerinnen und Bewohnern in vollstationäre Pflegeeinrichtungen. Ausnahmen wurden nur zugelassen, sofern eine 14-tägige Quarantäne in der Einrichtung sichergestellt werden konnte [13]. Hierzu sahen sich zunächst aber nur wenige Heime in der Lage.

In der Folge war es den Krankenhäusern vielfach nicht möglich, poststationäre Patienten, sei es nach durchgemachter COVID-19-Erkrankung oder nach anderen Behandlungsanlässen, zeitnah in die angestammte Pflegeeinrichtung zu entlassen oder einen neuen Aufnahmeplatz zu finden. Übergangsweise belegten die entsprechenden Patientinnen und Patienten ein Krankenhausbett oder wurden im Sinne einer Kurzzeitpflege in einer stationären Rehabilitationseinrichtung versorgt. Gegen beide Varianten wurden den ÄL-FüGK jedoch zunehmend Vorbehalte entgegengebracht, welche überwiegend mit dem Argument der nichtauskömmlichen Finanzierung begründet wurden. Dadurch ergab sich insbesondere in der Spätphase des Katastrophenfalls ein erheblicher Organisationsaufwand rund um das Management des postakuten COVID-19-Patientenkollektivs.

Im Verlauf lockerte das StMGP die Regelungen dahingehend, dass ausgeheilte COVID-19-Fälle vom Aufnahmestopp ausgenommen wurden sowie die 14-tägige Quarantäne auch schon während des Krankenhausaufenthalts durchgeführt werden konnte.

Am 25.05.2020 wurde der Aufnahmestopp durch die Verpflichtung zur Umsetzung entsprechender Hygienekonzepte in den Pflegeeinrichtungen ersetzt, sodass die Heimbewohnerschaft wieder an ihre angestammten Plätze zurückkehren konnten [12].

### Rückkehr zum Regelbetrieb/Stufenplan

Im Verlauf des April kam es zu einem Rückgang der täglichen Neuinfektionen sowie konsekutiv der Beanspruchung von Krankenhauskapazitäten durch COVID-19-Patienten (Abb. 2 und 4). Als logische Konsequenz passte die Staatsregierung ihre Strategie bezüglich der stationären Patientenversorgung an [7]. Beginnend mit dem 09.05.2020 wurde den Krankenhäusern in begrenztem Umfang die Durchführung planbarer Krankenhausbehandlungen wieder gestattet, mit der Maßgabe, 30 % ihrer Beatmungsbetten für die COVID-19-Behandlung zur Verfügung zu halten. Normalstationsbetten waren zu 25 % vorzuhalten, Betten in stationären Rehabilitationseinrichtungen zu 30 %. Allerdings mussten sich die Kliniken in die Lage versetzen, binnen 24 h zusätzliche 10 % sowie binnen 48 h nochmals 10 % ihrer Beatmungsbetten zur Verfügung zu stellen. Reine Privatkrankenhäuser waren bis auf wenige Ausnahmen von der Vorhalteverpflichtung befreit [7]. Bei der Berechnung der COVID-19-Vorhaltung waren jeweils die freien sowie die mit COVID-19-Fällen belegten Betten einzubeziehen und diese in Relation zur Bettenzahl am Stichtag 08.05.2020 zu setzen.

Gleichzeitig erteilte die Allgemeinverfügung den Bezirksregierungen die Ermächtigung, bei weiterer Entspannung der Infektionslage in Absprache mit dem ÄL-FüGK und mit Genehmigung der Krankenhausplanungsbehörde bis zu einer Untergrenze von 15 % vorzuhaltender Betten weitere Behandlungskapazitäten für die elektive Patientenversorgung freizugeben. Hiervon abweichend lag für Universitätsklinika die entsprechende Regelungskompetenz beim Bayerischen Staatsministerium für Wissenschaft und Kunst (StMWK) [7].

Aufgrund der zu diesem Zeitpunkt günstigen Entwicklung machte die Regierung der Oberpfalz am 28.05.2020 von dieser Möglichkeit Gebrauch und senkte die Vorhalteverpflichtung für die Oberpfälzer Kliniken auf 25 % der Beatmungsbetten sowie 15 % der Normalstationsbetten ab (Abb. 6; [25, 26]). Diese Entscheidung wurde nach intensiven Beratungen durch ein Gremium der zuständigen Stellen bei der Regierung, einschließlich des Präsidiums, und den ÄL-FüGK/dem ÄBRD getroffen. Für das Universitätsklinikum Regensburg erteilte das StMWK am 19.05.2020 die Erlaubnis, die Bettenvorhaltung auf Normalstationen auf 15 % zu reduzieren, während die Vorgabe zur Intensivbettenvorhaltung bei unverändert 30 % belassen wurde.

**Figure 7:**
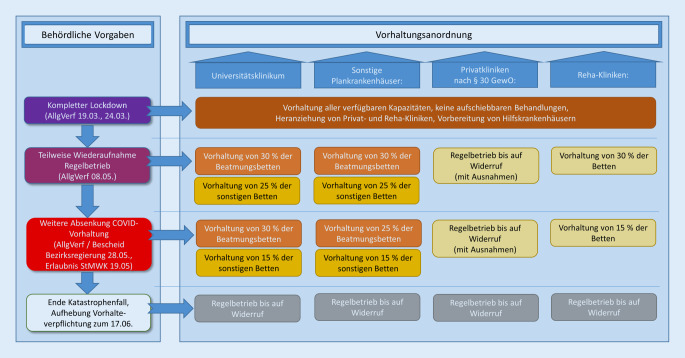

### Ende des Katastrophenfalls und Folgestrukturen

Mit Wirkung zum 16.06.2020 erfolgten die Aufhebung des Katastrophenfalls und damit die Auflösung der hierauf fußenden übergeordneten Strukturen des Bettenkapazitätsmanagements [4]. Gleichzeitig wurde die Vorhalteverpflichtung von COVID-19-Behandlungskapazitäten für die bayerischen Krankenhäuser aufgehoben [10, 27, 28].

Anstelle der ÄL-FüGK fiel die Rolle der Organisation der Krankenhausbelegung nunmehr den Pandemiebeauftragten der Kliniken im Zusammenwirken mit den ILS zu. Durch das Landesamt für Gesundheit und Lebensmittelsicherheit wurde eine regelmäßige Beratung zur Pandemielage etabliert. Die tägliche Meldepflicht der verfügbaren und COVID-19-belegten Krankenhauskapazitäten blieb unverändert bestehen [10]. Anstelle der FüGK richteten die betroffenen Staatsministerien je einen „Arbeitsstab zur Bewältigung großräumiger Gefährdungslagen und anderer koordinierungsbedürftiger Ereignisse“ ein. Die Kreisverwaltungsbehörden und Regierungen bildeten bei Bedarf Koordinierungsgruppen, deren Aufgaben weitgehend jenen der FüGK entsprach, ohne dass diese Gremien jedoch die Entscheidungsbefugnisse der jeweils zuständigen Behörden an sich ziehen konnten [2].

## Diskussion

Durch die SARS-CoV-2-Pandemielage sahen sich die Behörden genötigt, in einer bisher beispiellosen Anstrengung kurzfristig besondere Strukturen zur Krisenbewältigung zu konzeptionieren und umzusetzen. Binnen weniger Tage wurde im Rahmen einer komplexen Zusammenarbeit von Gesundheits- und Katastrophenschutzbehörden ein System zum übergeordneten Management von stationären Patientenströmen geschaffen. Die 3 für die Oberpfalz bestellten ÄL-FüGK zusammen mit dem ÄBRD erlebten sich dabei als Teil eines gut funktionierenden Steuerungssystems, sahen sich aber auch mit Problemen konfrontiert.

### Struktur- und Ablauforganisation

Das COVID-19-Bettenmanagement wurde in der Oberpfalz bewusst analog zu den im Tagesgeschäft üblichen und eingespielten Abläufen gestaltet. Dies entsprach weitgehend dem intuitiven Vorgehen der Krankenhäuser und ließ die Entscheidungshoheit der Kliniken für ihre individuellen Patientinnen und Patienten weitgehend unangetastet (z. B. Auswahl der Zielklinik innerhalb des Verlegungskorridors, Entscheidung über eine Übernahme je nach Krankheitsbild und eigenen Ressourcen durch die Zielklinik, Absprache des Verlegungszeitpunkts). Ferner konnte die Einschaltung einer weiteren Kommunikationsebene in Person des ÄL-FüGK hierdurch im Einzelfall vermieden werden. Uneingeschränkt bewährt hat sich dieses Vorgehen insbesondere bei zeitkritischen Notfallverlegungen, da eine Involvierung der ÄL-FüGK entfiel. Allerdings waren auch immer wieder Abweichungen vom skizzierten Konzept zu beobachten. Dieses Grundprinzip wurde mit wenigen Ausnahmen in ganz Bayern verwirklicht.

Geringe Schnittstellenprobleme gab es beim Verlegungsmanagement über Regierungsbezirksgrenzen hinweg, da ein benachbarter Bezirk die Patientenströme auf der Ebene von konkreten Einzelpatientinnen und -patienten über den ÄL-FüGK steuerte. Ungeachtet der positiven Erfahrungen, die dort mit diesem Ansatz gemacht wurden, kam es bei bezirksübergreifenden Absprachen zu einem erhöhten Koordinationsaufwand und zeitlichen Verzögerungen. Zudem musste zunächst ein datenschutzkonformer Modus bei der Übermittlung der Patientendaten gefunden werden. Für die Zukunft wäre in diesem Zusammenhang die Schaffung einer geschützten elektronischen Kommunikationsplattform für die ÄL-FüGK erstrebenswert.

In Bezug auf die innere Organisation wählten die Oberpfälzer ÄL-FüGK divergente Ansätze. Während mancherorts der ärztliche Leiter seine Aufgaben nur mit sporadischer Unterstützung durch Mitglieder der lokalen FüGK überwiegend allein erledigte, richtete andernorts ein ÄL-FüGK eine stabsmäßig organisierte Unterstützungsgruppe mit zeitweise täglichen Lagebesprechungen ein. Insbesondere bei der Abarbeitung von kurzfristig zu erledigenden, komplexen Auskunftsersuchen der FüGK Bayern hat sich die frühzeitige Einbeziehung von Unterstützungspersonal durch die ÄL-FüGK bewährt.

Da nicht für alle Aufgaben von ÄL-FüGK bzw. ÄBRD eine Vertretungsregelung vorgesehen war, muss bezweifelt werden, ob die Durchhaltefähigkeit dieses Konstrukts weit über das Ende der Katastrophenlage hinaus hätte für längere Zeit sichergestellt werden können.

### Betten- und Behandlungskapazitätenmanagement

Dank einer ausgeprägt kooperativen Zusammenarbeit der Krankenhäuser und des Rettungsdienstes konnte durch zeitnahe Verlegungen stets sichergestellt werden, dass alle Patientinnen und Patienten mit intensivmedizinischem Versorgungsbedarf unmittelbar heimatnah aufgenommen werden konnten. Der elektronische Bettenkapazitätennachweis IVENA Sonderlage hat sich beim Monitoring der Belegungssituation bewährt [19]. Neben der Bettenübersicht auf Ebene der Kliniken (teils sogar Abteilungen) erlaubt dieses Werkzeug auch die Berechnung der COVID-Ratio, welche als Steuerungsinstrument vorgeschlagen wurde [22]. Hierbei handelt es sich um das Verhältnis der mit COVID-19-Patienten belegten Intensivbetten zu den freien maximal betreibbaren Intensivbetten eines Krankenhauses oder einer Region.

Allerdings zeigte sich, dass die Koordinationsbemühungen der ÄL-FüGK immer wieder auf gegensätzliche Interessen aufseiten der Krankenhäuser stießen. Zum einen war eine gewisse Zurückhaltung zu beobachten, bei steigender Auslastung der Intensivkapazitäten diese durch frühzeitige Verlegungen zu entlasten. Bei der Wahl des Verlegungsziels tendierten die Kliniken zu Aufnahmekrankenhäusern mit einer höheren Versorgungsstufe, mutmaßlich u. a. dadurch begründet, dass die Transportkosten in diesen Fällen von den Krankenkassen getragen wurden. Vor allem aber wurde wiederholt der ausgeprägte Wunsch verschiedener Kliniken an die ÄL-FüGK herangetragen, postakute, entlassfähige COVID-19-Patientinnen und –Patienten aus den Akutkliniken abzuverlegen, obwohl jeweils in großem Umfang eigene Bettenkapazitäten vorhanden waren. Wiederholt wurden diese Beobachtungen offen mit finanziell motivierten Argumenten begründet. Vielfach kritisiert wurde auch die Verknüpfung der täglichen Bettenmeldungen der Krankenhäuser mit finanziellen Konsequenzen für diese, da eine Verzerrung der tatsächlichen Aufnahmekapazität befürchtet wurde.

Aus Sicht der Krankenhäuser ist dieses Verhalten durchaus konsequent: Als faktische Wirtschaftsunternehmen folgen diese erlösorientierten Prinzipien. In der konkreten Situation der SARS-CoV-2-Pandemie gilt es als Klinikträger folglich abzuwägen, ab welchem Zeitpunkt innerhalb des Zusammenspiels von DRG-Erlös und Ausgleichszahlungen für leer stehende Krankenhausbetten im Rahmen des COVID-19-Krankenhausentlastungsgesetzes das freie Bett profitabler ist als die Belegung mit einem COVID-19-Patienten [14].

Allerdings konnte der Abverlegungswunsch nicht länger krankenhauspflichtiger Patientinnen und Patienten in vielen Fällen nicht zeitnah erfüllt werden, da ein (Wieder‑)Aufnahmestopp in Pflegeeinrichtungen bestand. Zwar konnte in stationären Einrichtungen der Rehabilitation prinzipiell die notwendige 14-tägige Quarantäne in Form einer überbrückenden Kurzzeitpflege durchgeführt und abgerechnet werden [16, 17], die hierdurch für die Rehakliniken erzielbaren Erlöse seien nach Aussage der Betreiber jedoch nicht kostendeckend gewesen, sodass keine anhaltende Bereitschaft zur Übernahme von Kurzzeitpflegepatienten in größerem Umfang bestand.

Aus Sicht der ÄL-FüGK muss aus diesen Erfahrungen heraus die Forderung formuliert werden, im Rahmen einer evtl. zweiten Infektionswelle die Krankenhausrefinanzierung so zu gestalten, dass die resultierenden Anreize synergistisch mit den Steuerungsbemühungen der Patientenströme durch die Krisenstäbe wirken. Als hilfreich nahmen die Autoren die im Laufe des Katastrophenfalls abgegebene Zusage der Krankenkassen wahr, Verlegungskosten aus Kapazitätsgründen unabhängig vom Transportziel zu übernehmen [20].

### Ergänzende Ausstattung der Krankenhäuser

Zur ergänzenden Ausstattung zusätzlich geschaffener Intensivpflegebetten wurden von den Behörden Medizingeräte zur Verfügung gestellt, u. a. auch Beatmungsgeräte, die für die COVID-19-Versorgung vorgesehen waren. Unter den Beatmungsgeräten befanden sich u. a. auch Heimbeatmungsgeräte, welche nur bedingt oder gar nicht für den Einsatz in der Intensivmedizin geeignet, aufgrund des Turbinenprinzips und unzureichender Filter in infektiöser Umgebung nicht vor innerer Kontamination geschützt und durch die Krankenhäuser selbst nicht aufbereitbar waren. Keines dieser Geräte kam nach Information der Autoren in der Oberpfalz im Rahmen der COVID-19-Behandlung zum Einsatz. Retrospektiv betrachtet hätte der Aufwand zur Beschaffung derartiger Heimbeatmungsgeräte womöglich anderweitig größeren Nutzen erbracht.

### Stufenplan zur Wiederaufnahme des Routinebetriebs

Ende Mai hat die Regierung der Oberpfalz nach intensiven Beratungen mit den ÄL-FüGK mit einer zeitlichen Verzögerung von zweieinhalb Wochen von der Möglichkeit Gebrauch gemacht, die Vorhaltung von Krankenhauskapazitäten für die COVID-19-Versorgung weiter abzusenken. In Bezug auf den zeitlichen Ablauf und die Gestaltung der Vorhalteverpflichtung für Beatmungsbetten wurde ein eher konservatives Vorgehen beschlossen, während bei den Normalstationsbetten der volle Handlungsspielraum aus der einschlägigen Allgemeinverfügung ausgeschöpft wurde [7]. Angesichts einer zu diesem Zeitpunkt geringen oberpfalzweiten Belegung mit nichtbeatmungspflichtigen COVID-19-Patientinnen und -Patienten und einer um den Faktor 79 höheren Anzahl an freien Normalstations- und „Intermediate-care“-Betten hätte die Vorhalteabsenkung in diesem Segment sicherlich noch großzügiger ausfallen können.

### Limitationen

Die dargestellten Bettenkapazitätsdaten entstammen den täglichen Meldungen der einzelnen Krankenhäuser an die Katastrophenschutzbehörden. Diese waren nicht immer vollständig und konsistent. Zudem wurde zum 15.04.2020 die Meldeplattform gewechselt, und damit auch die Datenstruktur, was sich teilweise in Sprüngen der Parameter niederschlug. Zu beiden Abfragematrizes stand keine detaillierte Ausfüllanleitung zur Verfügung, sodass unterschiedliche Interpretationen der Datenfeldbezüge nicht ausgeschlossen werden können.

### Ausblick zweite Welle

Im Spätsommer 2020 manifestierte sich eine zweite COVID-19-Welle, sodass im Laufe des Novembers eine mit der ersten Welle vergleichbare COVID-Belegung der Krankenhäuser erwuchs. Das StMGP hat daher erneut ähnliche Steuerungsstrukturen geschaffen, allerdings mit leicht veränderter Terminologie [11]. Dabei verzichtete die Staatsregierung zunächst auf die Ausrufung des Katastrophenfalls, numerisch festgelegte Freihalteverpflichtungen für die Krankenhäuser und einen Aufnahmestopp für Pflegeeinrichtungen. Die bedarfsgerechte Einschränkung von elektiven Behandlungsfällen oblag zunächst den Kliniken selbst, konnte aber im Bedarfsfall auch vom ärztlichen Leiter angeordnet werden. Wie schon während der ersten Jahreshälfte wurden Ausgleichszahlungen für freistehende Krankenhausbetten indirekt mit den täglichen Bettenmeldungen der Kliniken verknüpft. Der weitere Verlauf bleibt abzuwarten.

## Schlussfolgerung

Während der ersten Welle der SARS-CoV-2-Pandemie fungierten die ÄL-FüGK und der ÄBRD als Verantwortliche für das übergeordnete Bettenkapazitätsmanagement und die Steuerung der stationären COVID-19-Patientenströme in der Oberpfalz. Intensivkapazitäten konnten durch die Krankenhäuser wirksam erhöht und lokalen Auslastungsspitzen durch gezielte Verlegungen begegnet werden. Es bestand lediglich geringer Bedarf an Normalstationsbetten bei einer historisch einmaligen Zahl an freien Betten. Insbesondere im Rahmen von Intensivverlegungen wurden gute Erfahrungen mit der Festlegung von Verlegungskorridoren unter weitgehender Aufrechterhaltung der Kommunikations- und Kooperationswege des Routinefalls gemacht. Der elektronische Krankenhauskapazitätennachweis IVENA Sonderlage leistete dabei wertvolle Dienste. Den Steuerungszielen gegenläufigen finanziellen Interessen der Krankenhäuser sollte für den Fall einer neuerlichen Krisensituation durch eine Neuregelung der COVID-19-Krankenhausrefinanzierung entgegengewirkt werden.

## Fazit für die Praxis

- Trotz im Bundesvergleich hoher Inzidenzraten konnte in der Oberpfalz durch geeignete Maßnahmen eine Überlastung der Krankenhauskapazitäten vermieden werden.
- Die Installation eines ärztlichen Entscheidungsträgers für die übergeordnete Steuerung der Patientenströme auf Rettungsdienstbereichsebene hat sich bewährt.
- Als positive Erfolgsfaktoren werden insbesondere die Definition von Verlegungskorridoren unter Beibehaltung etablierter Verlegungsprozesse und der elektronische Bettenkapazitätsnachweis gesehen.
- Überdacht werden sollten konkurrierende finanzielle Anreizsysteme seitens der Krankenhäuser, das Ausmaß der Vorhaltung von Normalstationskapazitäten und die Beschaffung bedingt geeigneter Medizingeräte.
